# Human endogenous retrovirus K and cancer: Innocent bystander or tumorigenic accomplice?

**DOI:** 10.1002/ijc.29003

**Published:** 2014-06-17

**Authors:** Ronan F. Downey, Francis J. Sullivan, Feng Wang-Johanning, Stefan Ambs, Francis J. Giles, Sharon A. Glynn

**Affiliations:** 1 Prostate Cancer Institute, National University of Ireland Galway, Galway, Ireland; 2 Department of Radiation Oncology, Galway University Hospitals, Galway, Ireland; 3 Center for Cancer and Metabolism, SRI International, Menlo Park, CA; 4 Laboratory of Human Carcinogenesis, National Cancer Institute, Bethesda, MD; 5 HRB Clinical Research Facilities Galway & Dublin, National University of Ireland Galway and Trinity College Dublin, Galway, Ireland

**Keywords:** human endogenous retrovirus, HERV-K, carcinogenesis, melanoma, breast cancer, prostate cancer, HERV-K activation, oncogenesis, immunomodulation, Env, Gag, Np9, Rec

## Abstract

Harbored as relics of ancient germline infections, human endogenous retroviruses (HERVs) now constitute up to 8% of our genome. A proportion of this sequence has been co-opted for molecular and cellular processes, beneficial to human physiology, such as the fusogenic activity of the envelope protein, a vital component of placentogenesis. However, the discovery of high levels of HERV-K mRNA and protein and even virions in a wide array of cancers has revealed that HERV-K may be playing a more sinister role–a role as an etiological agent in cancer itself. Whether the presence of this retroviral material is simply an epiphenomenon, or an actual causative factor, is a hotly debated topic. This review will summarize the current state of knowledge regarding HERV-K and cancer and attempt to outline the potential mechanisms by which HERV-K could be involved in the onset and promotion of carcinogenesis.

One of the most striking findings that arose from the publication of the human genome sequence was the enormous swathe of transposable elements (TEs) it harbored.^1^ Constituting ~45% of the entire sequence, they have co-evolved alongside the protein-coding component to contribute to modern-day phenotypes in ways which are still being deciphered. A subset of TEs, known as human endogenous retroviruses (HERVs), are ancestral relics of germline infections to which the genome succumbed over the course of evolution. The progenitors of these retroelements were exogenous retroviruses, which infected germline cells, subsequently became endogenized and subject to the laws of Mendelian inheritance.^2^ HERVs share the genomic structure universal to all retroviruses: 5′LTR-gag, *pro, pol, env*–3′LTR. In retroviruses, these open reading frames (ORFs) encode viral polyproteins, which, after post-translational modification, become the critical structural and functional proteins, such as the reverse transcriptase or the transmembrane envelope, while the long terminal repeats (LTRs) specify promoter, enhancer and polyadenylation signals.^3^ The vast majority of HERVs have acquired inactivating mutations such as stop codons or frameshifts, inhibiting the translation of functional proteins and thus making the production of a full, infectious retrovirus particle, from a single genetic locus, an impossibility.^4^

The HERV-K group is class II HERVs and exhibits closest homology to betaretroviruses, which cluster as class II retroelements. It consists of 11 subgroups (HML-1 to HML-11), each as the result of a separate germline infection.^5^ One of these subgroups, HML-2, has been subject to intensive research because it maintains an unrivalled coding competence with many of its proviruses maintaining complete, or near-complete, ORFs for all viral polyproteins (Fig. 1).^3^ Finally, it represents the most recently integrated HERV group into the human genome. Some HML-2 proviruses are both human specific and/or polymorphic indicating integration events subsequent to the human–chimpanzee split and within modern humans. This likely contributes to the fact that HML-2 is the least defective and most active retroviral family. In this regard, HML-2 is considered the most interesting HERV group to study in terms of potential oncogenic activity.

Overall, HML-2 is represented in the genome by 91 proviruses and 944 solitary (solo) LTRs. Solo LTRs are the result of unequal crossing over due to highly homologous sequences.^5^ Two main types of HERV-K (HML-2) are found in humans: type I is characterized by a 292 base pair deletion at the boundary of the *pol* and *env* (envelope) genes (Fig. 1), whereas type II lacks it. The deletion in type I proviruses leads to an alternative splicing event culminating in a protein known as Np9, while type II proviruses express a complete accessory protein known as Rec.^3^

HERVs play an important role in normal physiological function. For example, the protein syncytin 1 mediates cellular fusion of the placental trophoblast and is encoded by an env gene from the HERV-W group.^6^ Another syncytin— known as syncytin 2—plays a similar physiological role and is encoded by an env gene from the HERV-FRD group.^7^ Finally, the presence of HERVs, in particular their LTR elements, has added an additional layer of complexity to our genome, in that many of these LTRs have been co-opted by protein-coding genes and serve as regulatory elements directing tissue-specific expression.^8^

The association of HERVs with disease has garnered the most attention from researchers. HERVs have been implicated in autoimmune disorders,^9,10^ but with conflicting reports particularly involving multiple sclerosis (MS).^11–13^ Recent research refutes a role for deregulated HERV-W *env* in MS lesions, including the high-level-transcribed *ERVWE1* locus encoding Syncytin-1.^14^ In this review, we discuss the most recent developments in the field of HERV-K and human tumor biology, in particular emerging evidence of a role for HERV-K in immunomodulation and the presence of HERV-K in tumor-derived exosomes, further indicating the potentially important role of HERV-K in human carcinogenesis.

## HERV-K and Solid Tumors

To date there is evidence for HML-2 activation in ovarian cancer,^15,16^ melanoma,^17–19^ breast,^20–24^ prostate,^25–28^ lymphomas,^29^ leukemias^30^ and sarcomas.^31^ In the 1980s, Ono *et al*. successfully cloned HML-2, thanks to its similarity to mouse mammary tumor virus (MMTV).^32^ They also found that stimulation of human breast cancer cell lines with female steroid hormones led to an upregulation of HML-2 mRNA.^33^ Several groups followed with reports of HML-2 mRNA and viral particle expression in breast cancer.^29,34–36^ Wang-Johanning *et al*. refined this work to produce data that accurately quantified HML-2 *env* transcripts and spliced transcripts in breast tumors demonstrating elevated levels compared to unaffected controls.^20,21^ They also demonstrated an association between HML-2 Env protein expression in breast tumors and increased risk of lymph node metastasis and poor outcome in two separate US cohorts and a Chinese cohort of breast cancer patients,^37,38^ corroborating the findings of Golan *et al*.^23^ Most recently, Wang-Johanning *et al*. demonstrated that HML-2 serum mRNA and anti-Rec antibody titers are predictive of early-stage breast cancer. Additionally, HERV-K-gag copy number tended to be higher in breast cancer patients with a primary tumor who later on developed the metastatic.^39^

High levels of expression of HML-2 *env*, *rec* and *np9* mRNA, and Env protein have been reported in ovarian cancer cell lines and tumors,^16^ whereas in another study Np9 mRNA was not detectible in two ovarian tumors tested.^40^ One possible mechanism of altered HML-2 expression in ovarian cancer may be due to alterations in its methylation status.^15^

Retrovirus-like particles and the expression of HML-2 mRNA and proteins are detectable in prostate cancer tissues. Ishida *et al*. isolated a HML-2 Gag protein in the serum of a prostate cancer patient using serological recombinant cDNA expression cloning (SEREX) technology.^25^ They subsequently detected HML-2 *gag* mRNA in the serum of six of nine prostate cancer patients, but failed to detect HML-2 *gag* mRNA in LnCAP, DU145 or PC3 prostate cancer cells.^25^ Gene fusions are a frequent occurrence in prostate cancer, the majority of which involve the fusion of the transcription factors ETS translocation variant (ETV1) or ETS-related gene (ERG1), to the transmembrane protease, serine 2 (TMPRSS2). In these fusions, the androgen-responsive TMPRSS2 drives expression of the ETV1 or ERG1 oncogenes. Recently, ETV1-HERV-K fusions have been described, corresponding to the 5′-untranslated region (UTR) of HERV-K-22q11.23^26^ and HERV-K17.^41^ Additionally, the ETV1-HERV-K-22q11.23 fusion is also inducible in LNCaP in response to androgen,^26^ similar to HML-2 induction by estrogen and progesterone in breast cancer cell lines.^33^

Goering *et al.* detected significant expression of HERV-K-22q11.23 and HERV-K17 in the androgen-responsive prostate cancer cell lines 22Rv1, LNCaP and MDA-PCa-2b.^27^ Normal prostate cells and androgen-insensitive prostate cancer cells (PC-3, DU-145 and BPH-1) exhibited expression near the limit of detection.^27^ Expression of two other proviruses HERV-K-11q23.3 and HERV-K-22q.11.21 was not detectable in prostate cancer cell lines. Assessing HERV-K-22q11.23 5′UTR-*gag*, *env* and *Np9* gene expression in prostate tumors (*n* = 45) *versus* benign tissue (*n* = 11), the expression of the 5′UTR-*gag* and env region was significantly elevated in tumors compared to benign tissues. *Np9* was detectable only in a subset of carcinomas (18/45). In contrast, HERV-K17 was reduced in prostate tumors compared to benign. Where HERV-K-22q11.23 and HERV-K17 were expressed, they correlated with PSA levels, suggesting that HERV-K-22q11.23 and HERV-K17 retroelements are under androgen-inducible control, whereas HERV-K-11q23.3 and HERV-K-22q.11.21 are not.^27^ Wallace *et al*. demonstrated that the HERV-K *gag* mRNA in peripheral blood mononuclear cells (PBMCs) is predictive of diagnosis with prostate cancer and correlates with elevated plasma interferon-γ and IP10.^42^

## HERV-K and Hematological Malignancies

Brodsky *et al*. discovered a potential role for HERV-K in leukemia. They showed that HML-2 pol mRNA was expressed in the blood of patients suffering from chronic myeloid leukemia (CML) and acute myeloid leukemia (AML).^43,44^ Others also reported that HML-2 *gag* mRNA is present at higher levels in PBMCs of leukemia patients compared to healthy controls.^30^ Similar studies have reported HML-2 viral particles in lymphomas^29^ and HML-2 env expression in the H9 human T-cell lymphoma cell line.^45^ Additionally, the human lymphotropic herpesvirus Epstein–Barr virus (EBV), which has been implicated in the development of lymphoma, was shown to induce HERV-K18 *env* gene expression. The HERV-K18 env has been reported to have superantigen (SAg) activity by several groups,^46,47^ whereas others have found no evidence of SAg activity.^48,49^ Indeed, multiple HERV-K env proteins elicit antibody responses.^22,50^ A direct association between HERV-K18 env SAg and carcinogenesis has yet to be shown. HML-2 expression has also been seen to decrease after lymphoma therapy, indicating that it may be useful for monitoring therapeutic response.^29^

## HERV-K and Melanoma

The prevalence of HML-2 *pol, gag* and *env* mRNA, and Gag and Env proteins in melanoma is well established.^17–19,51–54^ In 2002, a sequence homologous to HERV-K (HML-6) was identified in melanoma patients (HERV-K-MEL).^31^ HERV-K-MEL, which produces an antigen spliced from a defective noncoding env-like ORF, was reported in cutaneous and ocular melanomas, and nevi. Antibodies raised against the HERV-K-MEL antigen were detectable in melanoma patients.^31^ Melanoma cell lines (SKMel-28, SKMel-1, 518A2, MelJuso, HS-Mel2 and JH-Mel6 and HV-Mel7), but not cultured melanocytes (NHEM neo 5935, NHEM neo 4528 and NHEM neo 6083), produce retrovirus-like particles that exhibit reverse transcriptase activity,^52^ which contain mature Gag and Env proteins. HML-2 Pol, Gag and Env,^52^ and accessory proteins Rec and Np9 have also been detected in melanoma.^18,51^ Further studies sought to predict the prognostic value of HERV-K in melanoma and found that HERV-K was a statistically significant marker of acrolentiginous, mucosal and uveal melanoma. Patients with serological response against HERV-K had a significantly decreased disease-specific overall survival.^55^ Additionally, HML-2 rec mRNA is expressed in melanoma cells but not in benign nevi or normal skin, indicating aberrant activation in melanoma. Furthermore, rec mRNA positivity correlated with the vertical growth phase of melanoma, a step that increases the risk of metastatic melanoma.^56^ A recent study by Schmitt *et al*. defined the HML-2 transcriptome in melanoma, identifying 23 different HML-2 loci as transcribed to varying degrees in different patient specimens and melanoma cell lines.^57^

## Polymorphic HML-2 Group Members

Of the 91 known HML-2 proviruses, 11 are polymorphic.^5^ The most recent insertions (~1 million years ago) include HERV-K-19p12 (K113) (29% of individuals) and HERV-K-8p23.1 (K115) (16% of individuals) as measured using a pool of mixed backgrounds.^58^ Other polymorphic HML-2 proviruses include: HERV-K-11q22.1 (K118), HERV-K-6q14.1 (K109), HERV-K-7p22.1a (K108R), HERV-K-8p23.1 (K115) and HERV-K-1p31.1(K116),^59,60^ in addition to HERV-K-3q13.2 (K106), HERV-K-7p22.1b (K108L), HERV-K-10p12.1 (K103), HERV-K-12q13.2 and finally HERV-K-U219 (K105) located in the unassembled centromeric region (Un_g1000219).^5^

It is currently not known whether inheriting specific HML-2 polymorphisms increases the risk of cancer. Burmeister *et al*. investigated the frequency of the polymorphic full-length HERV-K115 and HERV-K113 in 102 female breast cancer cases and 102 controls, but did not find a significant association with breast cancer (HERV-K-K113, 16.7 *vs*. 12.7%; HERV-K-K115, 4.9 vs. 9.8%). (Note the lower prevalence than reported above^58^ for both. This suggests ethnic differences in frequency of inheritance).^24^

## Mechanisms of HERV Activation and Regulation

The abundance of inactive HERVs present in our genome suggests that active, integrating proviruses are largely deleterious to the host. Novel intrinsic restriction factors exist which impede retroviral infection and some of these have the ability to target both exogenous and endogenous infections. APOBEC proteins can inhibit viral RNA, thus blocking their translation.^61^ Additionally, APOBEC3G can hypermutate and inactivate HERV DNA.^62^ Activation of these retroelements can therefore be an indication that cellular programs, crucial to a healthy phenotype, have gone awry.

A crucial question that needs to be addressed is whether activation of HERVs is simply an epiphenomenon or is necessary for disease progression? A large proportion of HERV loci have become silenced *via* DNA hypermethylation, an epigenetic phenomenon.^63^ Many cancers display a globally hypomethylated state^64^; thus, activation of HERVs during tumorigenesis may simply be a bystander effect of this epigenetic state. It has become increasingly clear that genomic instability, including deregulated transcription and genome plasticity, is enabled as a result of epigenetic changes that take place within tumors. Demethylation of specific HERV families, including HERV-W, HERV-K and HERV-H, has been reported in various cancers.^65^ Moreover, demethylation of TEs correlates with their transcriptional activation in prostate cancer.^27^ This indicates that where HERV transcription is increased in cancer cells, it is likely due in part to hypomethylation of their LTRs. HML-2 DNA hypomethylation has been reported in melanoma cell lines,^66^ prostate tumors^27^ and ovarian tumor.^15^ Interestingly, age was negatively associated with HML-2 methylation in PBMCs from healthy donors aged 20–88 years. The average onset of HML-2 methylation in PBMCs occurred at 40–63 years, implicating HML-2 DNA hypomethylation in aging.^67^ Another important epigenetic mechanism that influences transcriptional activity is histone modification, but the influence of histone methylation, acetylation or other modifications on HERV expression in malignancy is still unknown.

Known inducers of HML-2 *in vitro* include ultraviolet radiation in melanoma,^17,68^ hormones, including progesterone, estrogen and androgen in breast^20,33^ and prostate^26^ cancer cell lines and bone morphogenetic proteins and retinoic acid in testicular germ cell tumor cell lines.^69^ Urine from smokers has also been shown to lead to an increase in HERV expression in normal human dermal fibroblasts and urothelium *in vitro*.^70^ Other factors that may activate or be activated by HERV-K include infectious viruses such as EBV^71^ and human immunodeficiency virus (HIV-1),^72^ and transcription factors including NF-κB, NF-AT,^73^ MITF-M,^74^ Sp1,Sp3^75^ and YY1.^76^

## Possible Mechanisms of HERV-K-Induced Oncogenesis

### Insertional mutagenesis

**Figure 2.**
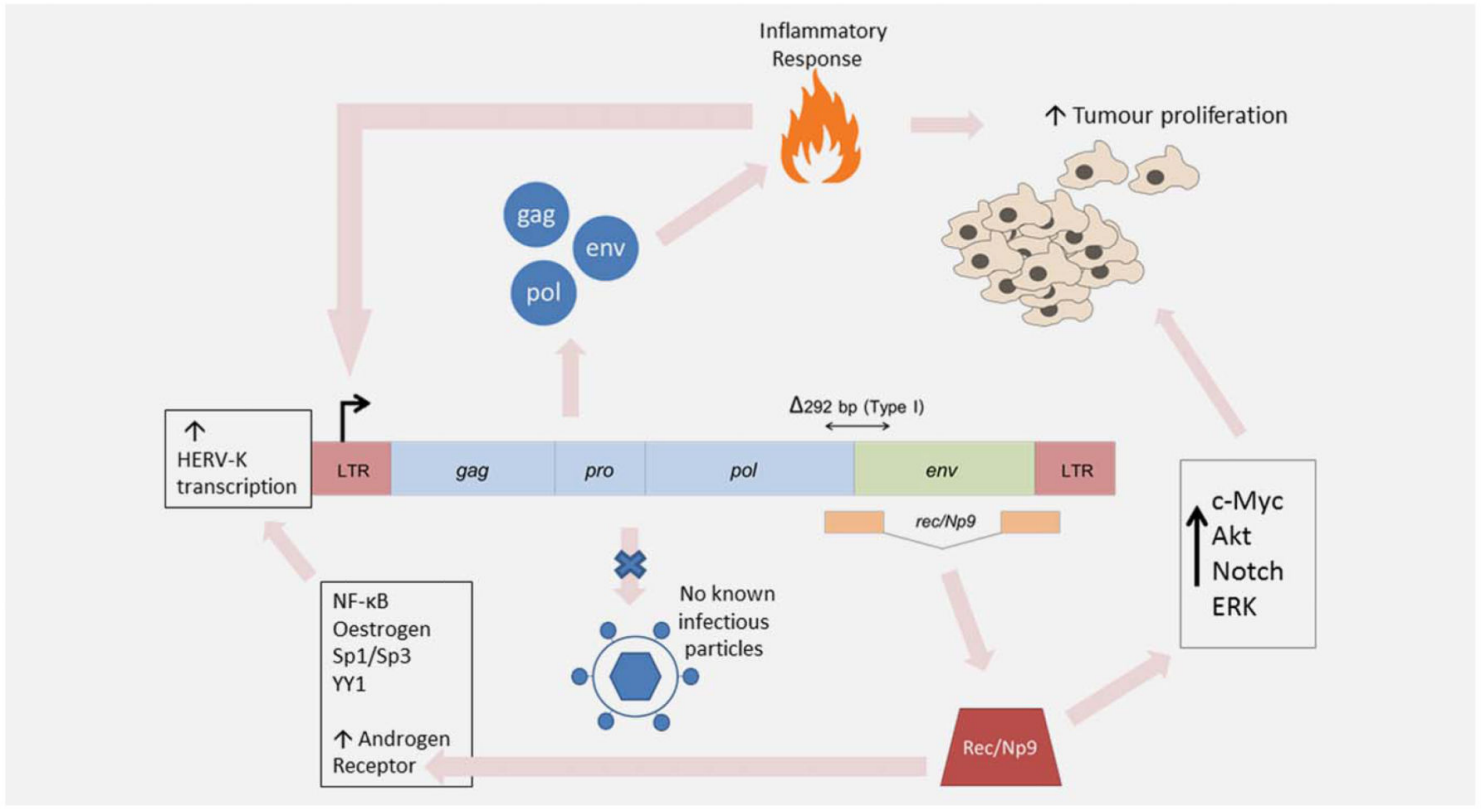
Proposed model of HERV-K (HML-2)-driven cancer progression. Global DNA hypomethylation during early-stage cancer leads to activation of otherwise silenced TEs, including HERVs. A humoral response to HERV-K gag has been observed in some cancers.^81^ Such a response to high levels of HERV-K protein expression may culminate in chronic inflammation. Conversely, it has been hypothesized that HERV-K LTRs are responsive to inflammatory transcription factors–a phenomenon that may explain the high levels of HERV-K mRNA and protein seen in some inflammatory diseases.^82^ HERV-K (HML-2) accessory proteins Rec and Np9 have been shown to lead to the derepression of the c-myc protooncogene,^83^ while Np9 has been shown to co-activate Akt, Notch and ERK pathways in leukemia.^84^ Rec has also been observed to lead to the derepression of the androgen receptor, which directly or undirectly causes a further increase in HERV-K transcription.^85^ Overall, the synergistic effects of chronic inflammation and dysregulated signaling/protooncogene activation caused by HERV-K protein expression may help to create a protumorigenic microenvironment culminating in further proliferation and metastasis. Finally, it is important to note that an active, infectious HML-2 provirus has not been isolated to date, but the existence of such a particle cannot be ruled out. It would potentially be oncogenic *via* mechanisms such as insertional mutagenesis.

HERVs may be oncogenic *via* insertional mutagenesis. However, to date, no fully intact and infectious HERV-derived retrovirus has been demonstrated *in vivo*. Retrovirus-like particles observed using electron microscopy in human placental trophoblasts,^77^ and teratocarcinoma^78^ and melanoma^52^ were identified as HERV-K derived. Efforts to identify an infectious HERV-K are compounded by the fact that the large majority are partially defective and also that a somatic integration event would be a relatively rare occurrence.^3^ Two independent groups have succeeded in resurrecting full retroviral particles after constructing consensus sequences representing ancestors of now defunct proviruses.^79,80^ Although these viruses were found to be only weakly infectious, these studies will prove invaluable in formulating hypotheses regarding the potential oncogenic mechanisms of an infectious HERV-K (Fig. 2).^83,85,88,122,123^

HERV-K113 and HERV-K115 are some of the most recently integrated HERVs in the human genome and represent obvious candidates for infectious proviruses. Boller *et al*. investigated this possibility and observed that HERV-K113 is able to produce fully intact retroviral particles *in vitro*.^86^ However, the authors concluded that an infectious HERV-K113 virus would be unlikely due to a lack of a functional reverse transcriptase.

### HERV-K Rec and Np9 as putative oncogenes

Rec exhibits functional homology to the Rev protein of HIV-1, a nucleocytoplasmic shuttle protein.^3^ Np9 is spliced from an alternative splice donor site to Rec, and shares only 14aa with Rec and Env, with no homology to Rev.^87^ Functional studies found that both proteins bind the promyelocytic leukemia zinc finger (PLZF) protein, a transcriptional repressor of the *C-MYC* proto-oncogene,^83^ leading to the derepression of C-MYC. Rec also binds a related protein known as testicular zinc-finger protein (TZFP), a transcriptional repressor of the AR. Rec inhibits the ability of TZFP to repress AR transcription.^88^ Hanke *et al*. identified an additional binding partner of Rec known as human small glutamine-rich tetratricopeptide repeat protein (hSGT), which also acts as a co-repressor of the AR.^85^ Moreover, they proposed a “vicious cycle” model, whereby increased cellular AR led to increased transcription at HERV-K loci and thus increased levels of Rec leading to further AR derepression. The involvement of such hormonal regulators will be interesting to study in castration-resistant prostate cancer, in which disruption of the AR signaling axis is a key factor in development of resistance.

Reinforcing the possible importance of these proteins in tumorigenesis was the finding that mice transgenic for the *Rec* gene are prone to seminomas.^89^ Np9 has been shown to interact with LNX—an E3 ubiquitin ligase that targets members of the NUMB/NOTCH pathway.^40^ This pathway has been implicated in the regulation of proliferation of cancers of the breast and prostate.^90^ Finally, a recent study has shown that Np9 acts as a critical molecular switch for co-activating β-catenin, ERK, Akt and Notch1 and promoting the growth of human leukemia stem/progenitor cells (Fig. 2).^84^

### HERV-K-induced immunomodulation

In a Darwinian sense, cancerous tissue uses the inflammatory-associated milieu of the tumor microenvironment to confer a selective advantage.^91^ The apparent immunogenicity of HERV proteins therefore represents a potential contributor to, or initiator of a chronic inflammatory state, beneficial to tumor survival (Fig. 2). HML-2 antibodies have been observed in patients with melanoma,^18^ breast^39^ and ovarian cancers.^16^ In breast cancer, studies have found that both humoral and cell-mediated immune responses to HERVs were enhanced in patients when compared to controls.^22^ HERV-K18 Env protein has been shown to elicit T-cell responses and can be upregulated in response to EBV infection,^46,92^ and may be a prerequisite of B-cell lymphomas.^93^

Similar to discoveries in HIV-1, HERV-K may encode env proteins with immunosuppressive transmembrane domains. A recent study by Morozov *et al*. identified an immunosuppressive HERV-K env protein, which altered cytokine expression and suppressed immune cell proliferation *in vitro*.^94^

Nitric oxide (NO) is an endogenous free radical signaling molecule that has been intimately linked with inflammation, wound healing responses and cancer.^95,96^ A significant association between nitric oxide synthase 2 (NOS2) and HML-2 Env expression has been demonstrated in breast cancer.^37^ NOS2 is an independent predictor of poor outcome in estrogen receptor-negative breast cancer, associated with macrophage infiltration, deregulated p53 signaling, increased proliferation and resistance to apoptosis.^95,97,98^ Can HML-2 Env proteins mediate downstream inflammatory effects *via* their activation of NO signaling? Intriguingly, β-catenin, ERK and Akt, which are activated by Np9,^84^ are also activated by NO signaling.^98,99^

### Exosomes

An evolving hypothesis in cancer research over the last few years has been the involvement of tumor exosomes in metastasis.^100,101^ Exosomes are nanoscale membrane vesicles that are secreted from cells and are thought to be important intercellular communicators, or, in a cancer setting, drivers of metastatic spread.^102^ A recent study has now implicated HERVs in this process, with the finding that HML-2 mRNA is selectively packaged into tumor exosomes and that this genetic material can be transferred to normal cells.^103^ The authors also demonstrated that these exosomes were enriched for the *C-MYC* protooncogene, which has been shown to be regulated by PLZF, a target of Rec and Np9.^83^ Therefore, it is possible that there is a link between the high levels of HERV-K mRNA and *C-MYC* in these exosomes, but further investigation will have to be done in this regard. Another important point is that HML-2-driven metastasis *via* exosomes would not require an envelope gene, as exosomes gain entry to target cells *via* a plasma membrane fusion event. In essence, exosomes could potentially empower the abundance of defective HERVs with a new-found infectivity.^104^

### HERV-K viral proteins as potential vaccines

Although the direct oncogenic effects of HERVs in cancer remain to be fully elucidated, there is potential for their use as diagnostic or prognostic biomarkers and for immunotherapeutic purposes including vaccines. Independent groups have demonstrated a strong association between HERV-K antibodies and clinical manifestation of disease and therapeutic response.^23,29^ Antibodies recognizing synthetic HERV-K proteins were detected at a very low frequency in the sera of healthy donors.^16,22^ Humoral anti-HERV-K immune response may provide additional prognostic information to that of established melanoma markers.^31,55^ Data from these studies reveal a significant inverse correlation between serological anti-HERV-K reactivity and patient survival probability in melanoma patients. Among the different classes of tumor antigens recognizable by the immune system, mutated self-antigens and viral antigens are unique because they are foreign to the host and not subjected to preexisting antigen-specific tolerance.^105–107^ HML-2 exons coding for mature proteins are spread out over the genome and are a repository of immunogenic retroviral gene products that can be “reawakened” when genetic damage occurs through chromosome breaks, frameshifts and mutations, removing sequences normally silencing protein expression.

HERV-K MEL is an antigenic peptide that is encoded by a short ORF from a processed HERV-K (HML-6) pseudogene and has been shown to be recognized by cytotoxic T cells in human melanoma.^31^ BCG, vaccinia and yellow fever vaccinations are associated with a reduced risk of developing melanoma,^108–110^ although conflicting data exist for yellow fever vaccines.^111^ It is suggested that this effect is due to antigen sequence homology between these vaccines and HERV-K-MEL leading to cross-reaction between vaccine-elicited cytotoxic T cells and melanoma cells.^112^ Reintroduction of these vaccines has been suggested as a novel method of melanoma immunoprevention; otherwise, HERV-K MEL represents a legitimate target for cellular immunotherapy.^112,113^

## Future Perspectives

Over the course of evolution, our genome has been locked in a molecular “war” with exogenous infectious agents. Ultimately, it is this very battleground, together with viral endogenization, which has bestowed upon us the diverse genetic repertoire we possess today. Constituting 8% of our genome, these HERVs have supplied us with an additional layer of plasticity and physiological functionality, yet scientists now believe that hidden detrimental processes fueled by HERVs may be present, which are inducing chronic diseases such as cancer and autoimmunity. As of yet, no truly infectious HERVs have been observed. However, as outlined in this review, a range of potential molecular mechanisms involving the retroviral proteins may be aiding and abetting both tumor formation and metastasis. Ultimately, it is likely that many of these mechanisms are working synergistically to produce these effects, and the heralding of a single molecular event induced by a HERV protein is improbable.

Ascribing a causative role for a particular agent to a disease has long been a challenging task. Criteria such as Hills criteria^114^ and Koch’s postulates^115^ have been formulated to address this problem. These criteria have recently been refined and built upon in light of HERVs-postulated role in human disease.^113,116,117^ However, even if a direct link between HERVs and carcinogenesis is never established, their presence may be highly advantageous in terms of the implementation of novel biomarkers for cancer. Further work will need to effectively correlate their presence with various disease stages and also make the necessary comparisons against “gold standard” biomarkers. Equally promising is the potential to take advantage of tumor-specific HERV expression for the use of targeted immunotherapies. Wang-Johanning *et al*. have demonstrated the potential of anti-HML-2-Env antibodies in inhibiting tumor growth and inducing apoptosis, both *in vitro* and in *in vivo* mouse xenograft models.^37^ This work represents a major milestone in research into HERVs and cancer and it is likely that targeting Env in a similar fashion in other cancers will be equally effective. However, it remains imperative that these studies are evaluated in a clinical setting. Additionally, it may also be possible to conjugate these antibodies to cytotoxic drugs for increased effect.^118^ Similarly, Kraus *et al*. demonstrated that HML-2-Env-targeted vaccine reduced renal tumor metastasis in a murine model.^119^ Novel therapies, such as these, are key to making inroads toward a future cure for the increasingly complex and multistep disease that is cancer. However, their safety must be assessed given the newly established role of HERV-K in embryonic stem cells and iPS cells,^120^ which may have implications for pregnancy. Their role in adult stem cells is not currently known.

Several limitations exist in the field of cancer-related HERV-K research, including a lack of adequately powered patient population studies to determine the role of HERV-K in the etiology of cancer, and/or its association with metastasis, therapeutic response and overall patient survival. A gap exists in our knowledge as to which HERV-K loci are specifically activated in cancer. A recent study by Schmitt *et al*. has defined the HML-2 transcriptome in melanoma, identifying 23 loci as transcribed,^57^ and it is an imperative that similar studies be initiated in other cancers. A causal role for HML-2 has yet to be established. Generally, retroviruses induce tumours by insertional mutagenesis targeting specific oncogenes, as is the case with HBV.^121^ This is an unlikely mechanism though in the case of HML-2. Evidence does suggest that Rec and Np9 may be putative oncogenes, but whether Gag or Env are also oncogenic is not known. In exceptional cases such as Jagsiekte sheep retrovirus (JSRV) the Env protein has been found to be causal (ovine pulmonary adenocarcinoma).^122^ However, it is unlikely that HML-2 Gag or Env have a similar causal effect; potentially they may influence carcinogenesis by activating or perturbing inflammation responses against cancer.

It is our belief that within the next decade these genetic “squatters” will have firmly established themselves within the modern multistep model of cancer progression and their expression will be viewed as an “enabling characteristic” of cancer, giving new meaning to the famous words of Nobel laureate J. Michael Bishop when he stated that “the seeds of cancer are within us”.^123^

## Figures and Tables

**Figure 1.**
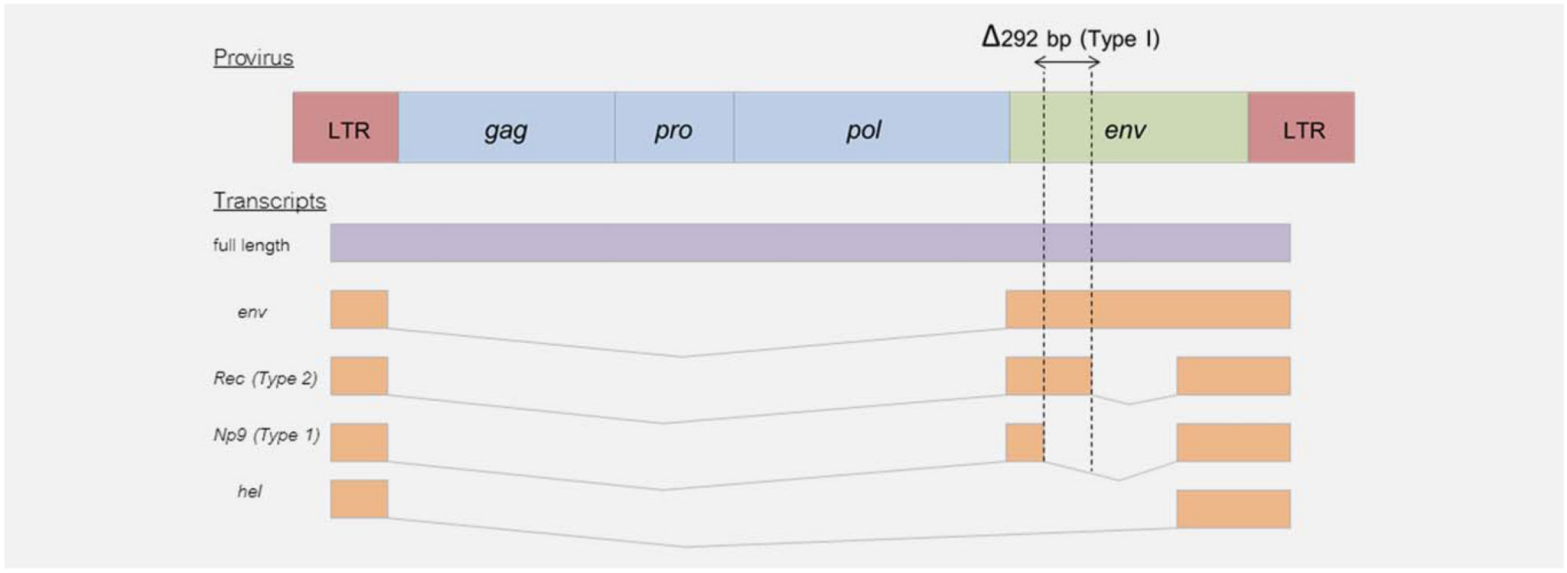
Structure of HERV-K provirus. The full length (gag) HML-2 transcript encodes the gag, pro and pol polyproteins. A singly spliced transcript encodes the env polyprotein, while a doubly spliced transcript encodes either the Rec or Np9 accessory proteins depending on the presence or absence of a 292-bp deletion at the pol/env boundary–a characteristic that defines a HML-2 provirus as either Type 1 (deleted) or Type 2 (intact). HML-2 also expresses a 1.5-kb transcript of unknown function known as the hel transcript.

## Abbreviations:

AML: acute myeloid leukemia
APOBEC3G: apolipoprotein B mRNA-editing, enzyme-catalytic, polypeptide-like 3G
AR: androgen receptor
CML: chronic myeloid leukemia
EBV: Epstein-Barr virus
eMLV: ectropic murine leukemia virus
ERG1: ETS-related gene
ETV1: ETS translocation variant
HERV: human endogenous retrovirus
HIV-1: human immunodeficiency virus
HPV: human papillomavirus
hSGT: human small glutamine-rich tetratricopeptide repeat protein
HTLV-1: human T-lymphotropic virus
LTR: long terminal repeats
MHC: major histocompatability complex
MMTV: mouse mammary tumor virus
NO: nitric oxide
NOS2: nitric oxide synthase 2
ORF: open reading frames
PBMC: peripheral blood mononuclear cell
PLZF: promyelocytic leukemia zinc finger
Sags: superantigens
SEREX: serological recombinant cDNA expression cloning
TE: transposable element
TLR: toll-like receptor
TMPRSS2: transmembrane protease, serine 2
TZFP: testicular zinc-finger protein
